# m6A Regulators Mediated Methylation Modification Patterns and Tumor Microenvironment Infiltration Characterization In Nasopharyngeal Carcinoma

**DOI:** 10.3389/fimmu.2021.762243

**Published:** 2022-01-07

**Authors:** Zijian Liu, Jinlan He, Jiaqi Han, Jiangping Yang, Wenjun Liao, Nianyong Chen

**Affiliations:** ^1^ Department of Head and Neck Oncology, Cancer Center and State Key Laboratory of Biotherapy, West China Hospital, Sichuan University, Chengdu, China; ^2^ Department of Radiation Oncology, Cancer Center and State Key Laboratory of Biotherapy, West China Hospital, Sichuan University, Chengdu, China

**Keywords:** m6A score, nasopharyngeal carcinoma, molecular subtype, tumor microenvironment, TIDE, immunotherapy

## Abstract

**Background:**

The role of RNA N6-methyladenosine (m6A) modification in tumor progression and metastasis has been demonstrated. Nonetheless, potential biological function of m6A modification patterns in nasopharyngeal carcinoma (NPC) remains unknown.

**Methods:**

The m6A modification patterns were comprehensively evaluated based on 26 m6A regulators in NPC, and m6A subtype and also m6A score were identified and systematically correlated with representative tumor characteristics.

**Results:**

Two distinct m6A subtypes were determined and were highly consistent with immune activated and immune suppressed phenotypes, respectively. More representative m6A scores of individual tumors could predict tumor microenvironment (TME) infiltration, mRNA based stemness index (mRNAsi), EBV gene expression, genetic variation, and prognosis of NPC patients. Low m6A score, characterized by activation of immunity and suppression of mRNAsi and EBV gene, indicated an activated TME phenotype and better PFS and also lower risk of recurrence and metastasis. High m6A score, characterized by activation of Wnt and NF-κB signaling pathway and lack of effective immune infiltration, indicated an immune suppressed TME phenotype and poorer survival. Low m6A score was also correlated with increased tumor mutation burden (TMB) and better response to immunotherapy, and vice versa. A significant therapeutic advantage in patients with low m6A score was confirmed with an anti-PDL1 immunotherapy cohort.

**Conclusions:**

m6A patterns played an important role in the diversity and complexity of TME. m6A score could be used to evaluate the m6A pattern of individual tumor to enhance our understanding of TME infiltration and guide more effective immunotherapy strategies.

## Introduction

Nasopharyngeal carcinoma (NPC), deriving from squamous epithelial cells of the nasopharyngeal mucosa, is the most common malignant tumor in the head and neck and endemic in South China and Southeast Asia (1). With the use of intensity-modulated radiotherapy (IMRT) and development of comprehensive therapeutics, namely, concurrent and induction chemotherapy, loco-regional control has been greatly improved while distant metastasis has become the predominant cause of treatment failure in NPC (2). Biologically, an Epstein–Barr virus (EBV) infection is deemed as a primary etiological factor for NPC (3), and this virus-associated cancer represents the typical “inflamed tumor” which exhibits abundant infiltrating immune cells in the tumor microenvironment (TME) (4). These features make immunotherapy a promising treatment for NPC patients. However, unlike the remarkable efficacy of immune checkpoint inhibitors (ICIs) in other types of tumor (5), the response to ICIs is unsatisfying in NPC patients (6, 7). Moreover, the EBV status and TME characteristics are significantly heterogeneous in NPC (8), raising a question of whether these differences lead to the distinct risks of immunotherapy responses. To further improve the efficacy of immunotherapy in NPC, it is quite urgent to analyze the TME to better identify the mechanism underlying the low response to ICIs.

N6-methyladenosine (m6A) modification, the most prevalent RNA modification in eukaryotic cells, is a dynamic and reversible process regulated by methyltransferases (“writers”), demethylases (“erasers”), and RNA binding proteins (“readers”) (9). m6A modification can regulate lots of physiological and pathological activities, namely, circadian rhythm, stress response, neural differentiation, learning, obesity, infertility, and carcinogenesis (10–12). Accumulating evidence has indicated that m6A modification could interplay with immune system and influence the infiltration of immune cells. The deletion of RNA binding protein YTHDF1 can significantly enhance the antitumor immune response of antigen-specific CD8^+^ T cells, and PD-L1 checkpoint blockade can induce complete regression of tumors in YTHDF1-deficient mice (13). Demethylase FTO significantly promoted melanoma tumorigenesis and decreased response to anti-PD-1 blockade immunotherapy, suggesting that FTO inhibition combined with anti-PD-1 blockade may sensitize immunotherapy in melanoma (14). A recent study has also shown that m6A modification played a critical role in the formation of TME diversity and complexity in gastric cancer, and this expression pattern could reflect the characterization of TME infiltration and the effectiveness of immunotherapy (15). Nevertheless, the function of dominant immune cells remains uncertain in NPC and whether these immune cells are regulated by the mechanism of m6A modification still needs to be investigated. Therefore, it is worth exploring whether m6A modification could influence immune cells infiltration and immunotherapy response in NPC.

A recent study has shown that the risk signature composed of three m6A related genes (IGF2BP1 + IGF2BP2 + METTL3) was an independent prognostic factor and could predict the clinicopathological features of NPC (16). However, it was confined to only a few m6A regulators and patient samples owing to limitations of analytical methods. With the emergence of more analytical techniques, methods such as single-sample gene set enrichment analysis (ssGSEA) are applied to reanalyze these data to calculate the relative amount of infiltration immune cells in each sample (17). Meanwhile, there are numerous genomic and transcriptomic sequencing data revealing the driver mutations, aberrant regulation, and disease subtypes in NPC. Hence, we conducted integrated bioinformatics with the genomic information and clinical traits of NPC samples from public database to comprehensively evaluate the m6A modification subtypes and also the corresponding TME characteristics correlated with each subtype. A m6A score was further constructed to predict the response to immunotherapy.

## Materials and Methods

### Dataset Source and Preprocessing

The raw data of gene expression and DNA methylation were downloaded from the database of Gene Expression Omnibus (GEO) (https://www.ncbi.nlm.nih.gov/geo/), and the basic information of involved datasets are summarized in **Table S1**. In total, 10 eligible NPC datasets were gathered. Raw microarray data downloaded as the “CEL” files were normalized and analyzed, while high throughput sequencing data were directly downloaded. For DNA methylation data, the normalized average beta values and annotation for the probes were directly downloaded. The results of somatic mutation and copy number variation were directly downloaded from supplementary materials of Zhang and his colleagues’ work (18), and the corresponding definitions of characteristics of the patient, namely, TME subtypes, morphology, and EBV gene expression were also adopted. The immunotherapeutic cohort of metastatic urothelial cancer patients treated with an anti-PD-L1 antibody atezolizumab (IMvigor210 cohort) was used as the validation cohort (19), and the expression data and detailed clinical annotations were obtained from http://research-pub.Gene.com/imvigor210corebiologies based on the Creative Commons 3.0 License. Moreover, the fragments per kilobase million (FPKM) values and clinical information of pan-cancer involving 17 cancer types in the Cancer Genome Atlas (TCGA) database were downloaded from the UCSC XENA database (https://xenabrowser.net/datapages/) (20).

### Differentially Expressed Gene and Gene Ontology (GO) Analysis

The empirical Bayesian approach of “limma” R package was applied to conduct a differentially expressed gene analysis between different defined groups and the significance criterion was set as adjusted P-value <0.05 and log_2_FC >1. The differentially expressed mRNAs were visualized in heatmap and volcano plot in R using R package “pheatmap” and “ggplot2”. The candidate genes were imported into The Database for Annotation, Visualization and Integrated Discovery (DAVID) v6.8 (21) to conduct GO analysis, mainly consisting of biological process and the Kyoto Encyclopedia of Genes and Genomes (KEGG) pathways.

### Unsupervised Clustering for m6A Regulators

A total of 26 m6A regulators were extracted from published articles identifying different m6A modification patterns involved in NPC (15, 16, 22), namely, 10 writers (KIAA1429, WTAP, RBM15, RBM15B, ZC3H13, METTL3, METTL5, METTL14, METTL16, and CBLL1), 14 readers (YTHDC1, YTHDC2, YTHDF1, YTHDF2, YTHDF3, HNRNPC, FMR1, IGF2BP1, IGF2BP2, IGF2BP3, RBMX, HNRNPA2B1, LRPPRC, and ELAVL1), and 2 erasers (FTO and ALKBH5). An algorithm nonnegative matrix factorization (NMF) based on decomposition by parts was used to identify distinct m6A modification patterns according to the expression of 26 m6A regulators and progression free survival (PFS) of patients (23). The “CancerSubtypes” package was used to perform the NMF clustering with 1,000 repetitions, guaranteeing the stability of classification. The number and stability of clusters were determined by p-value of survival analysis and the value of average silhouette width, respectively. The identification of m6A gene cluster was also conducted in the same way with relevant parameters.

### Implementation of ssGSEA

To calculate single sample gene set enrichment, the GSEA program was used to derive the absolute enrichment scores of validated gene signatures. In brief, the enrichment scores of both biological process and infiltration immune cells were quantified by ssGSEA in R package “GSVA (Gene Set Variation Analysis)”, a non-parametric and unsupervised method to estimate the variation of gene set enrichment (24). The gene sets of “c5.all.v6.2. symbols” downloaded from the MSigDB database and another gene set with genes connected to some biological processes (19) were both utilized to run GSVA analysis to predict underlying biological function. Furthermore, the relative abundance of each immune cell infiltration in the NPC tumor microenvironment was also conducted using ssGSEA algorithm. Meanwhile, for the sake of rigorousness, two sets of independently published immune cell markers, namely, immune cell signature 1 and 2, were involved in our study, containing 24 and 23 types of immune cells respectively (17, 25). Due to its wide use, signature 1 was the main immune cell signature in the whole study for assessing the enrichment level of immune cell infiltration, while signature 2 was used as the validation and supplement. To roughly assess EBV gene expression in NPC samples, genes significantly correlated with EBV genes [Pearson coefficient >0.3 reported before (18)] were extracted for ssGSEA algorithm as the profile of EBV gene expression was not available in the database. All the above gene sets and immune cell markers are summarized in **Tables S2**-**5**.

### Identification of m6A Gene Signature and Calculation of m6A Score

Differentially expressed genes (DEGs) were obtained from different m6A clusters and the overlapped DEGs among the clusters were extracted and regarded as m6A gene signature. m6A gene clusters were also conducted using this signature with unsupervised clustering, after which the prognostic analysis for each gene in the signature was performed using univariate Cox regression model. Genes with significant prognostic value (p-value <0.01) were extracted for calculation by principal component analysis (PCA), while PC1 and PC2 of each sample were calculated by using the expression matrix of genes with prognostic significance (gene i) (15). The m6A score was calculated as follows:


$$m6A\;score\;=\;\Sigma \left(PC1_{i}+\;PC2_{i}\right)$$

### Prediction of Immunotherapy Response of Patients

To predict the immunotherapy response of NPC patients, the Tumor Immune Dysfunction and Exclusion (TIDE) database (http://tide.dfci.harvard.edu/) was referred to, with which multiple published transcriptomic biomarkers could be estimated to predict immunotherapy response (26). The TIDE value was calculated to assess the probability of immunotherapy response, and the cutoff value of TIDE was defaulted as 0. As the input data should be normalized and the recommended tumor types for this database were melanoma and non-small cell lung cancer (NSCLC), the results could only play an auxiliary role in this study.

### Calculation of Gene Expression Based Stemness Index for Patients

To assess the stemness of cancer cells, the gene expression based stemness index (mRNAsi) was calculated with the instruction of a one-class logistic regression algorithm for each NPC sample (27). The mRNA expression-based signature contained a gene expression profile composed of 11,774 genes, and the workflow to generate the stemness index was available on a previously established database (https://bioinformaticsfmrp.github.io/PanCanStem_Web/). Stemness index model using Spearman correlation was applied to score the NPC samples and the stemness index was subsequently mapped to the [0,1] range *via* utilizing a linear transformation that subtracted the minimum and divided by the maximum (27).

### Statistical Analysis

Correlation coefficients and p-values were conducted by Spearman correlation analysis among several defined groups. Single cell analysis was conducted on website (db.cngb.org/npcatlas) (28) while the expression level of LRPPRC in each cell and the images of distribution were downloaded directly from the same website and further processed in Adobe Illustrator. Kruskal–Wallis tests were used to compare differences among more than two groups, and Wilcoxon tests were used to compare differences between two groups. The “surv-cutpoint” function of the “survminer” R package was applied to search the best cutoff value in survival analysis. According to the best cutoff value, defined groups could be divided into high or low expression groups for further analysis. The Kaplan–Meier method was used to generate the survival curves and log-rank tests were utilized to identify significance of differences. Univariate Cox regression model was used to calculate the hazard ratios (HR) for candidate input genes. The waterfall function of “maftools” package was applied to present the mutation landscape of patients in NPC samples. All statistical p-values were two-sided, with p <0.05 as statistically significant. All data processing was performed in R 4.0.3 software.

## Results

### Landscape of m6A Regulators in Nasopharyngeal Carcinoma

**Figure 1:**
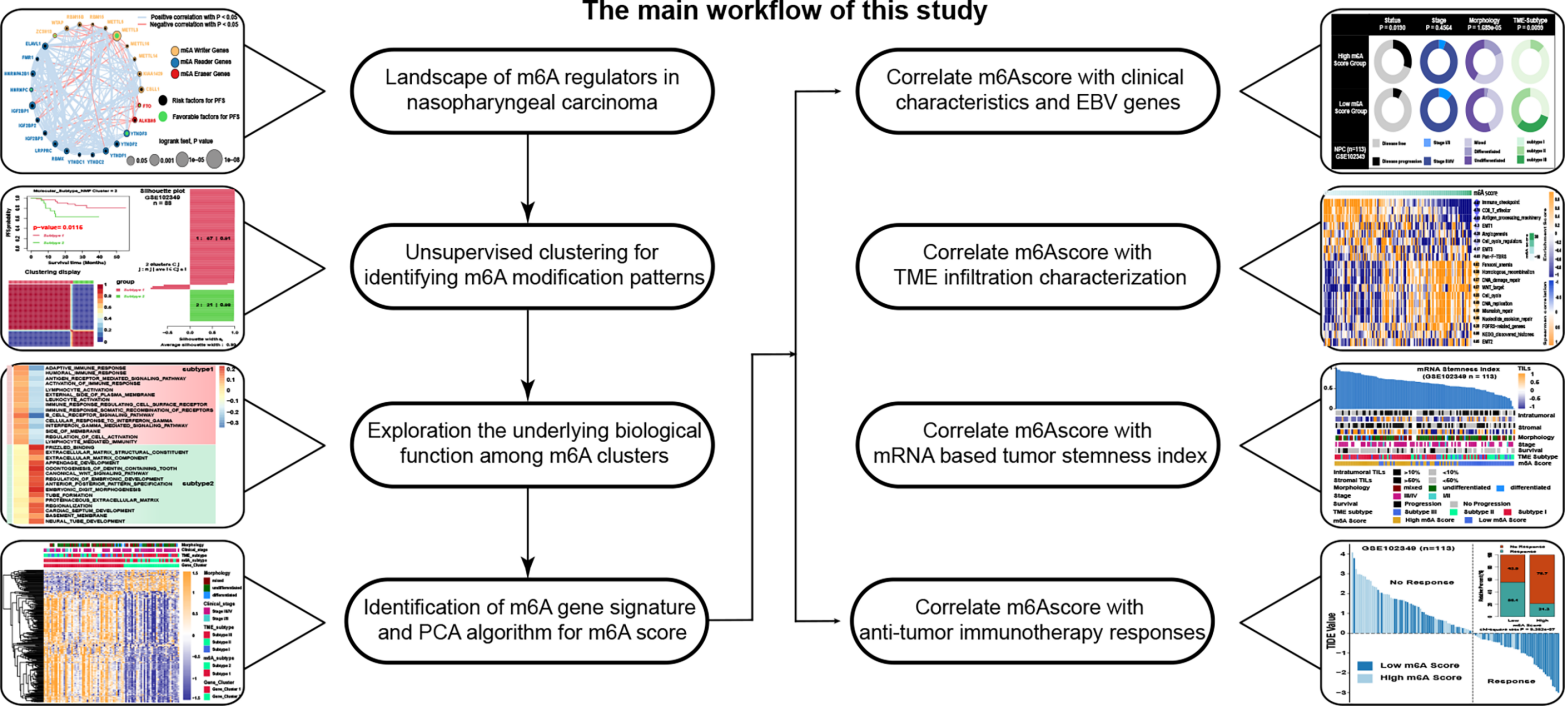
The main workflow of this study.

**Figure 2:**
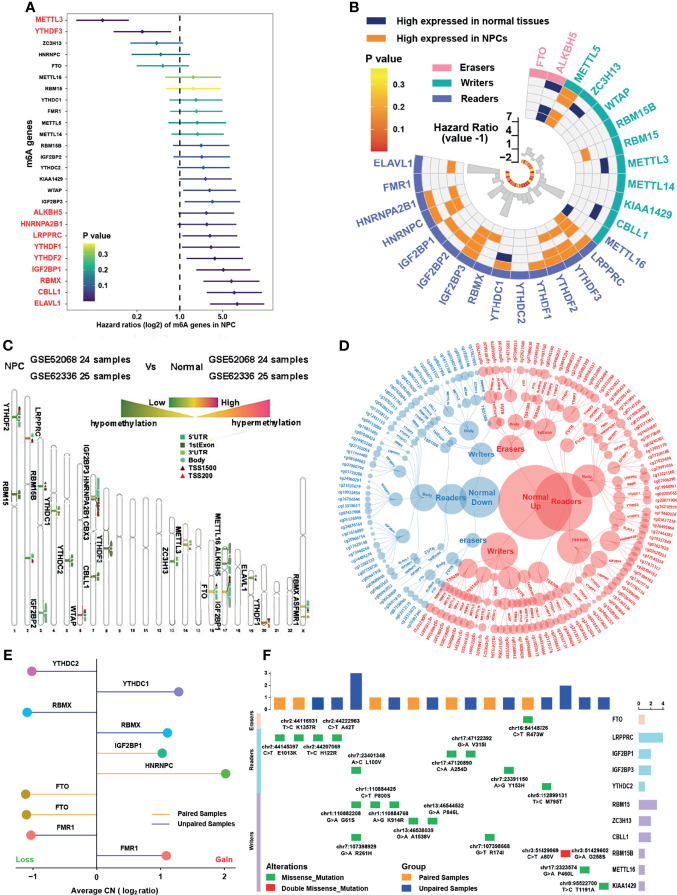
Landscape of m6A regulators in nasopharyngeal carcinoma. **(A)** The prognostic analyses for 26 m6A regulators in GSE102349 cohorts, and hazard ratio >1 indicated risk factors for survival and hazard ratio <1 indicated protective factors for survival. **(B)** Differentially expressed m6A regulators in NPC samples compared with normal tissues in five independent NPC datasets in GEO database, namely, GSE64634, GSE12452, GSE13597, GSE34573, and GSE53819. The inner columns represented HR values and P values of m6A regulators for PFS in GSE102349. **(C)** Average DNA methylation difference level of m6A regulators between NPC samples and normal tissues in two independent GEO datasets (GSE52068 and GSE62336). The degree to which methylation sites differ for each m6A gene was labeled on the chromosome. The darker red color indicated higher methylation and the darker green color indicated lower methylation. **(D)** Specific DNA methylation sites of m6A regulators and the red dot represented hypermethylation in normal tissue while the blue dot represented hypermethylation in NPC. The categories of each site were also shown. **(E)** Copy number variation of m6A regulators in NPC in GSE102349 dataset. **(F)** The somatic mutations of m6A regulators in NPC in GSE102349 dataset. Paired cohort referred to samples with matched blood samples and unpaired cohort referred to tumor samples only.

A total of 26 m6A related regulators from previous studies (15, 16, 22) were involved, namely, 10 writers, 14 readers, and 2 erasers. The main workflow is presented in **Figure 1**. To reveal the landscape of m6A regulators between NPC and normal nasopharyngeal tissues, we investigated the differential distribution of mRNA expression, DNA methylation, copy number variation, and somatic mutation of m6A regulators by integrated bioinformatics analysis. Firstly, survival analysis in 88 NPC patients with PFS data from GSE102349 indicated that METTL3 and YTHDF3 were protective factors while 9 m6A regulators (ALKBH5, HNRNPA2B1, LRPPRC, YTHDF1, THDF2, IGF2BP1, RBMX, CBLL1, and ELAVL1) were risk factors for PFS (**Figure 2A**). Then, differential gene expression analysis conducted in 5 GEO datasets (GSE64634, GSE12452, GSE13597, GSE34573, and GSE53819) between NPC samples and normal control samples revealed that most of the m6A regulators were highly expressed in NPC with the criterion of log_2_FC >1 and FDR >0.05 (**Figure 2B** and **Figure S1A**). To further investigate the upstream mechanism of changes in gene expression, genetic variations of m6A regulators were analyzed. DNA methylation analysis conducted in GSE62336 and GSE52068 indicated that the methylation level for most of the m6A regulators was lower in NPC samples than that in normal samples, suggesting that a lower DNA methylation level might be the reason for a higher mRNA expression in NPC (**Figure 2C**). Principle component analysis (PCA) showed there was significant distinction existing in the m6A DNA methylation between cancer and normal tissues (**Figure S1B**). The differentially expressed DNA methylation sites of m6A regulators and their correlated counterpart reference CpG island types are displayed in **Figure 2D**. By reanalyzing the published mutational and somatic copy number landscape of NPC (18), we spotted copy number variations (**Figure 2E**) and somatic mutations (**Figure 2F**) but just in a few samples. In 57 samples with matched blood samples, only 7 were with somatic mutations and 4 with copy number variations in m6A regulators. The LRPPRC was the most frequently mutated reader among all NPC samples with 4 mutational sites, while RBM15 was the most frequently mutated writer with 3 mutational sites. The above results showed a high heterogeneity in the expression and DNA methylation of m6A regulators between NPC and normal tissues, indicating the crucial role of m6A regulators in the occurrence and progression of NPC.

### Identification of m6A Subtypes and Biological Function Analysis

**Figure 3:**
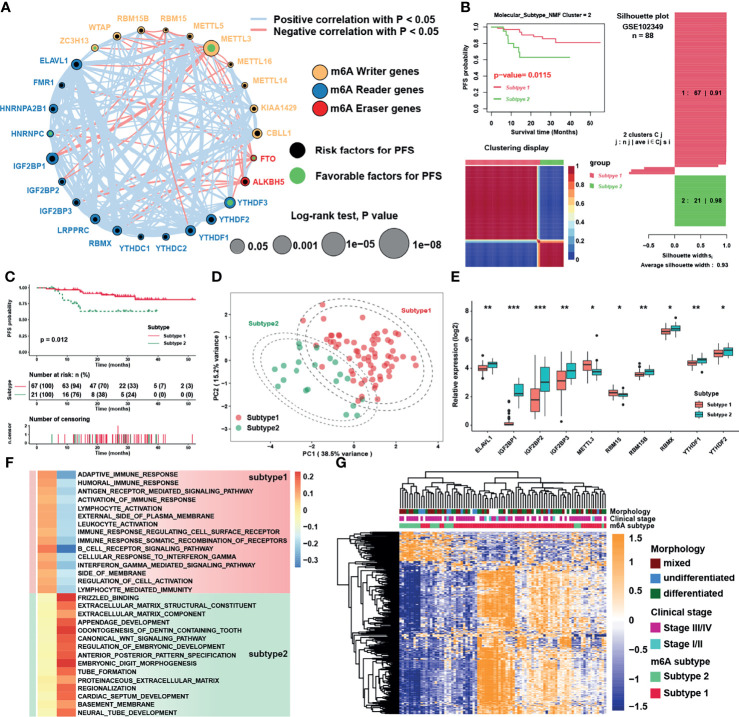
Identification of m6A subtypes and biological function analysis. **(A)** The interaction among m6A regulators in NPC. The circle size represented the effect of each regulator on the prognosis, and the range of values was calculated by Log-rank test. Black dots and green dots in the circle represented risk and favorable factors of prognosis, respectively. The lines linking regulators showed their interactions, and thickness of lines showed the correlation strength. Negative correlation was marked with red and positive correlation with gray lines. **(B)** The result of NMF algorithm for k = 2 with the best average silhouette width 0.93. **(C)** Survival analysis for m6A subtypes in NPC. **(D)** Principal component analysis for the transcriptome profiles of m6A subtypes. The dashed circles represented 95% confidence interval. **(E)** Significantly different m6A regulators between two m6A subtypes. **(F)** GSEA analysis revealed distinct enriched gene sets between subtypes. In the heatmap, rows showed the selected 30 gene sets, and columns showed consensus scores for each subtype. **(G)** Heatmap of differentially expressed genes between subtypes 1 and 2, and m6A subtypes, clinical stage and morphology were used as patient annotations. The asterisks represented the statistical p-value (*P < 0.05; **P < 0.01; ***P < 0.001) in panel **(E)**.

The comprehensive landscape of the interactions and connections among the m6A regulators and also the prognostic significance of m6A regulators in NPC patients in GSE102349 were illustrated with the m6A regulator network (**Figure 3A** and **Table S6**). A remarkable correlation in expression was found between m6A regulators not only in the same functional category but also across three categories. In particular, negative correlations were mostly shown between erasers (FTO, ALKBH5) and other categories, while METTL3 was the most significantly negatively correlated with IGF2BP1 among all correlations. To further explore the expression patterns among the m6A regulators, NMF algorithms were applied to construct two m6A subtypes combining the expression of m6A regulators and PFS of patients, namely, subtype 1 and subtype 2 (**Figure 3B**). The results showed that the survival curves (**Figure 3C**) and m6A transcriptional profile expression patterns of the two m6A subtypes (**Figure 3D**) were significantly distinctive. In addition, the m6A regulators were significantly differentially expressed between subtypes (**Figure 3E**). To explore the underlying mechanism of m6A subtypes, GSEA with distinct enriched gene sets was conducted (**Figure 3F**). The results indicated that immune-related pathways were highly activated in subtype 1 while tumor progression biological processes were highly activated in subtype 2. Differential expression analysis was used to identify representative genes before conducting GO analysis and further verifying the results of GSEA analysis. Indeed, we determined 310 m6A phenotype-related DEGs between m6A subtypes (**Figure 3G**, **Figure S1C** and **Table S7**), among which differentially expressed genes between subtypes were highly enriched in Wnt related signaling pathways and immune related pathways (**Figure S1D**). Taken together, the results confirmed that m6A modification might play an important role in immune regulation and cell proliferation in tumor microenvironment.

### Immune Cell Infiltration Characteristics in Distinct m6A Subtypes

**Figure 4:**
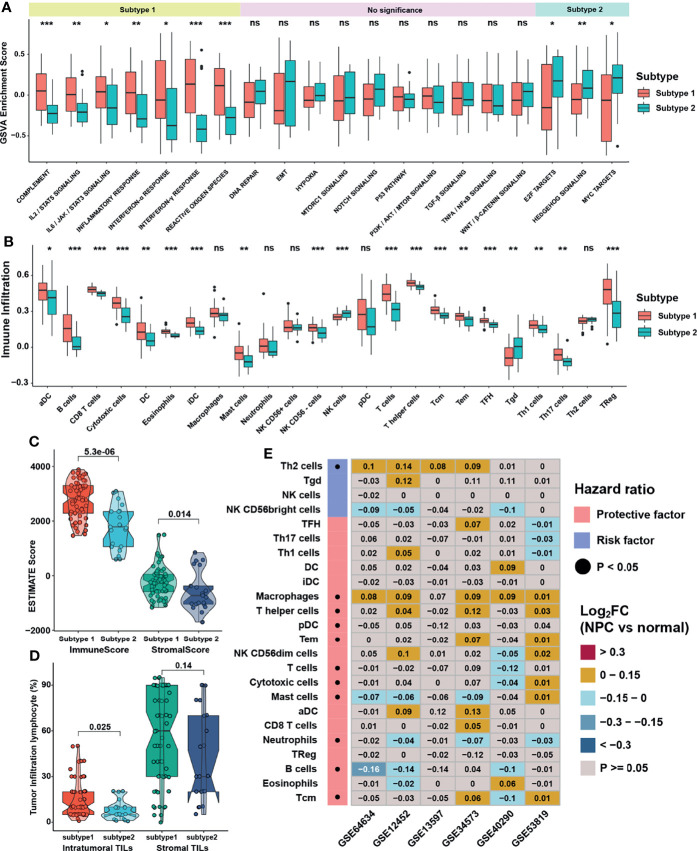
Immune cell infiltration characteristics in distinct m6A subtypes. **(A, B)** The abundance of GSVA score for curated pathways and TME infiltration cell in m6A subtypes. The upper and lower ends of the boxes represented interquartile range of values. The lines in the boxes represented median values, and black dots showed outliers. **(C)** Differences in immune and stromal scores *via* ESTIMATE between m6A subtypes. **(D)** Differences in intratumor and stromal tumor infiltration lymphocytes provided in GSE102349 between m6A subtypes. **(E)** Differentially expressed analysis for TME infiltration in 6 independent datasets, and black dots in left columns represented P < 0.05. The asterisks represented the statistical p-value (*P < 0.05; **P < 0.01; ***P < 0.001) in panels **(A, B)**. ns, no significance.

GSVA was performed to investigate the biological function between two distinct m6A subtypes. As displayed in **Figure 4A**, m6A subtype 1 was markedly enriched in immune response signaling pathways such as interferon-α/γ, IL-6/JAK/STAT3, and complement pathways, while m6A subtype 2 presented enriched pathways associated with E2F, MYC, and Hedgehog pathways. ssGSEA was then applied to calculate relative expression level of immune cells with specific immune cell signatures, and the result showed that no matter using immune cell signature 1 or 2, subtype1 was significantly rich in immune cell infiltration, namely, dendritic cells, myeloid cells, T cells and B cells (**Figure 4B** and **Figure S2A**). These results reflected immune pathways were activated and immune cells were highly enriched in m6A subtype1. Using Estimation of Stromal and Immune cells in malignant tumors using Expression data (ESTIMATE) method to infer the fraction of stromal and immune cells in tumor samples, stromal and immune cells scores were found to be higher in subtype 1, which were consistent with the TME infiltration analysis (**Figure 4C**). The percentage of tumor infiltration lymphocyte provided in a previous study (18) was also compared. Although the average percentage of stromal lymphocytes was not apparently different between m6A subtypes, that of intra-tumoral lymphocytes was significantly higher in subtype1 (**Figure 4D**). To further verify the influence of immune cell infiltration on PFS, survival analysis was conducted and showed that only the infiltration level of Th2 cell was a significant risk factor for PFS, while the central memory T cell (Tcm) was the most significant protective factor (**Figure S2B**). Thus, we analyzed the infiltration level of immune cells between NPC and normal samples in 6 NPC datasets to investigate the dominant cells in TME of NPC. To our surprise, only the infiltration level of Th2 cells was significantly higher in NPC samples, whereas that of B cells, the main target cells of EBV in the initiation of NPC, was consistently lower in NPC samples (**Figure 4E**). Subsequent exploration on the relative expression of EBV genes in GES102349 dataset using ssGSEA indicated that differences indeed existed between m6A subtypes but lacked consistency (**Figure S2C**). Furthermore, correlation analysis between m6A regulators and immune cell infiltration levels was conducted to identify key m6A regulators associated with immune response (**Figure S2D**), and the result showed that LRPPRC, HNRNPA2B1, and ELAVL1 were significantly negatively correlated with the infiltration level of Th2 cell but positively correlated with protective immune cells infiltration such as Tcm, B cells, and T cells. Combining the results of differential gene expression and survival analysis, LRPPRC, which was highly expressed in NPC samples and a risk factor for PFS, was regarded as a candidate m6A regulator in NPC. Finally, single cell analysis for LRPPRC was conducted in a published database [28]. Results showed that LRPPRC were highly expressed in cancer associated fibroblasts (CAFs), epithelial cells, and malignant NPC cells (**Figure S2E**), indicating that LRPPRC might negatively correlate with the activation and recruitment of immune cells, and positively correlate with the transition processes from epithelial cells to malignant cells or even tumor metastasis.

### Construction of m6A Score and Functional Annotation

**Figure 5:**
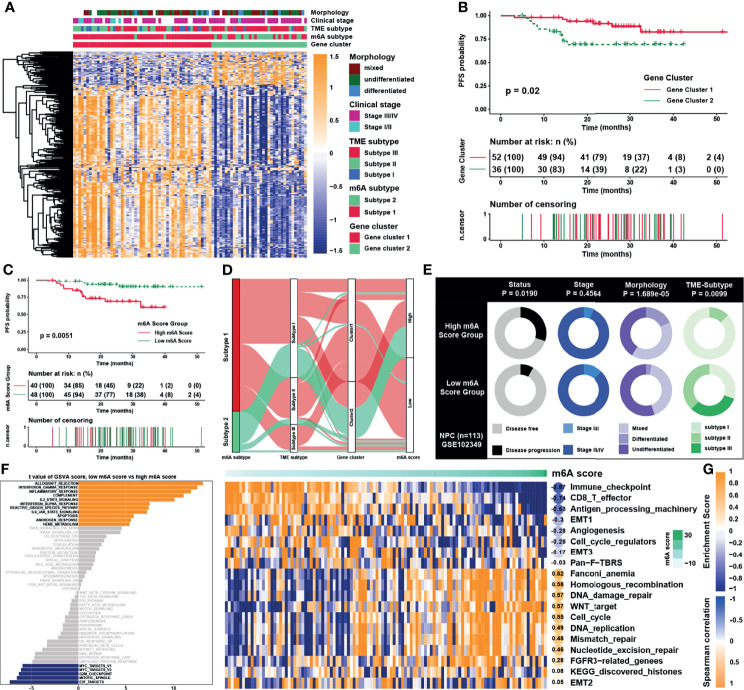
Construction of m6A score and functional annotation. **(A)** An unsupervised clustering of overlapped m6A phenotype-related genes in GSE102349 to classify patients into different genomic subtypes, termed as m6A gene clusters 1 and 2, respectively. The gene clusters, m6A subtypes, TME subtypes, clinical stage, and morphology were used as patient annotations. **(B)** Survival analysis for m6A gene cluster in GSE102349. **(C)** Survival analysis for m6A score in GSE102349. **(D)** Alluvial diagram showing the changes of m6A subtypes, m6A gene clusters, TME subtypes, and m6A score. **(E)** Clinical characterization in low and high m6A score groups, and the chi-square test was used to calculate statistical differences. **(F)** The abundance of GSVA score for curated pathways in two m6A score groups. The orange and blue columns represented pathways enriched in low and high m6A score group, respectively. **(G)** Correlations between m6Ascore and the known gene signatures in NPC using Spearman analysis. Negative and positive correlations were marked in blue and orange, respectively.

As 310 m6A phenotype-related DEGs had been identified between m6A subtypes, unsupervised clustering analyses were then performed based on the expression of these phenotype-related genes and PFS of patients to classify patients into more specific genomic clusters. Two distinct m6A modification genomic phenotypes named as m6A gene clusters 1 and 2 were revealed (**Figure S3A** and **Table S8**). Results showed that the m6A gene clusters were more representative than m6A subtypes, because the former had a higher average silhouette width of 0.94 and the number of samples between groups was more balanced. The heatmap (**Figure 5A**) and survival analysis (**Figure 5B**) reflected that gene clusters reshaped by m6A clusters displayed better consistency in gene enrichment than the m6A subtypes. Given the individual heterogeneity and complexity of m6A modification, a set of scoring system termed as m6A score was constructed based on 115 phenotype related genes with significant prognostic value (P <0.01) to quantify the m6A modification level of individual patients with NPC. Patients were then divided into low or high m6A score group by “survminer” package, and patients with low m6A score presented a prominent survival benefit than those with high m6A score (**Figure 5C**). The visualization of attribute changes of individual patients using alluvial diagram (**Figure 5D**) indicated that m6A score might be the best method to display individual m6A level. To better clarify the characteristics of m6A signature, we examined the correlation between the m6A score and the previously defined TME signatures and clinical traits (**Figure 5E** and **Figures S3B–C**). The m6A scores were lower in low m6A score group, m6A gene subtype 1 and m6A cluster 1, with all these groups displaying better prognosis. As for clinical traits, m6A scores were lower in stage I/II group, TME based subtype II/III, and undifferentiated histological type. The expression of m6A regulators was also significantly distinctive between low and high m6A score groups (**Figure S3D**). To further verify the underlying biological function elucidated above, GSVA was carried out whose results showed that the low m6A score group was enriched in interferon response pathways and immune related pathways such as IL-2/STAT5, IL-6/JAK/STAT3, while the high m6A score group was significantly associated with E2F, G2M, and MYC related pathways (**Figure 5F**). Furthermore, another pathway signature (19) was applied to verify the correlation between m6A score and the enrichment score of specific pathways (**Figure 5G**), indicating that m6A score was negatively correlated with immune related pathways while positively correlated with Wnt signaling pathway, DNA damage repair, and homologous recombination.

### Characteristics and Biological Function of m6A Score in NPC

**Figure 6:**
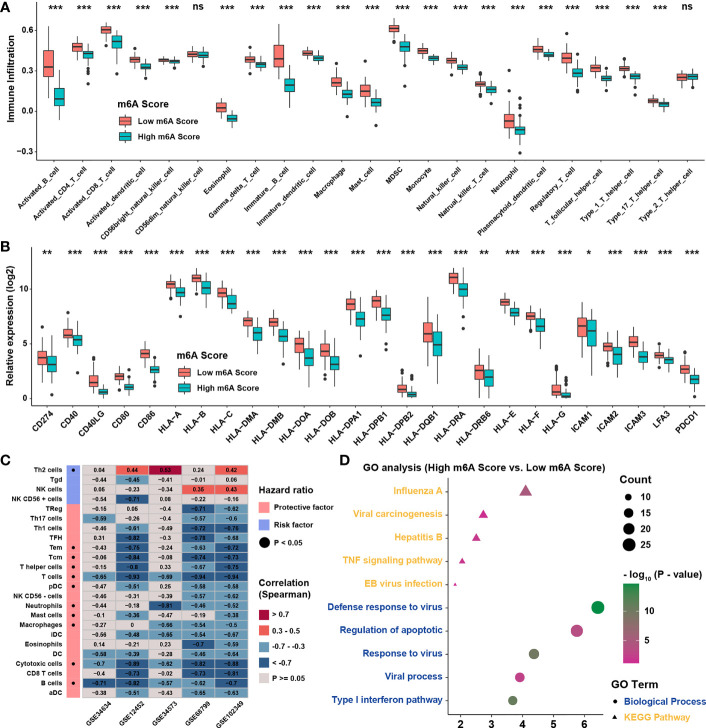
TME infiltration characteristics of m6A score. **(A)** The abundance of TME infiltration cell assessed by immune cell signature 2 in different m6A score groups. **(B)** The abundance of MHC molecules, costimulatory molecules, and adhesion molecule in different m6A score groups. **(C)** Correlations between m6A score and TME infiltration in 5 independent NPC datasets using Spearman analysis. **(D)** GO analysis for differentially expressed genes between m6A score groups. *P < 0.05; **P < 0.01; ***P < 0.001; ns, no significance.

**Figure 7:**
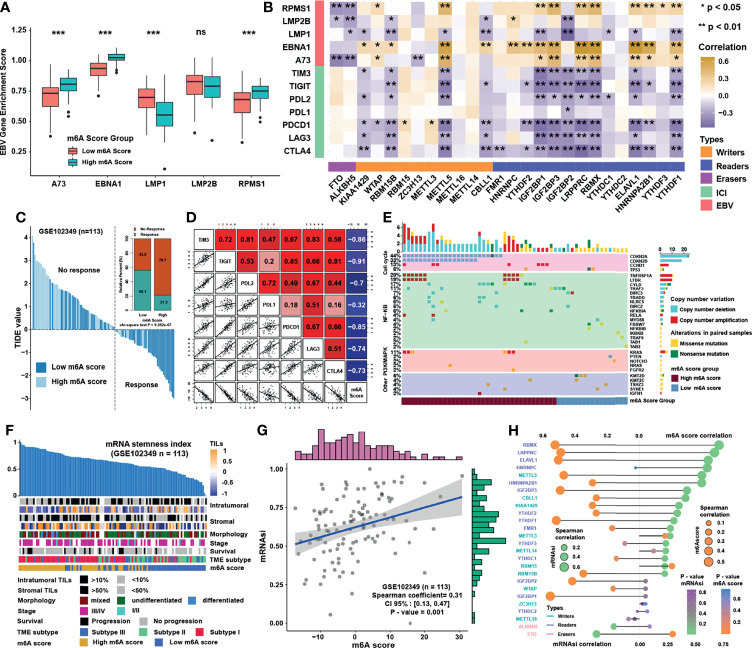
Characteristics and biological functions of m6A score in NPC. **(A)** The abundance of EBV gene enrichment score in m6A score groups. **(B)** Correlations between m6A regulators and immune checkpoint molecules and EBV gene GSVA score in GSE102349 using Spearman analysis. **(C)** TIDE value of NPC samples in GSE102349 was shown in different m6A score groups and the chi-square test was used to calculate statistical differences. **(D)** Correlation analysis of m6A score and immune checkpoint inhibitors. **(E)** Mutation and somatic copy number variations for the paired NPC cohort in GSE102349 and m6A score groups for each patient. **(F)** An overview of the association between known clinical and molecular features and mRNAsi in NPC. Columns represented samples sorted by mRNAsi from low to high (top row). Rows represented known clinical and molecular features. **(G)** Correlation analysis between mRNAsi and m6A score using Spearman analysis. **(H)** Correlation analysis between mRNAsi and m6A score and m6A regulators using Spearman analysis. The dot size represented the correlation level, and the color represented P-value. *P < 0.05; **P < 0.01; ***P < 0.001; ns, no significance.

Previous study showed that dendritic cells (DCs) were the key professional antigen-presenting cells responsible for the activation of naive T cells, and the activation of DCs depended on the high expression of MHC molecules, costimulatory factors, and adhesion factors (29). ssGSEA applied with immune cell signature 2 showed that activated DCs, immature DCs, and plasmacytoid DCs were all highly enriched in the low m6A score group (**Figure 6A**). Moreover, most of the MHC molecules, costimulatory molecules, and adhesion molecules were highly expressed in low m6A score group (**Figure 6B**). Given the great potential of m6A score in predicting immune response in NPC, m6A scores were calculated in 5 NPC datasets and the correlation with immune cell infiltration levels enriched was conducted by ssGSEA with immune cell signature 1 (**Figure 6C**). The result showed that m6A score was positively correlated with Th2 cells but negatively associated with CD8 T cells and DCs. Therefore, GO and KEGG analyses were conducted for the differentially expressed genes between two score groups, whose results revealed that high m6A score group was enriched in viral carcinogenesis and viral process (**Figure 6D**). Interestingly, the expressions of EBV genes such as A73, EBNA1, and PRMS1 were significantly higher in high m6A score group (**Figure 7A**), indicating that the worse prognosis and lower immune cell infiltration level might be correlated with EBV infection. Furthermore, we tested the correlation between m6A regulators and expression level of EBV genes as well as immune checkpoint molecules (**Figure 7B**). As expected, most of m6A regulators had positive correlation with EBV genes and negative correlation with checkpoint molecules, suggesting low m6A score group might have better response to immunotherapy and high m6A score group might have higher risk of recurrence and metastasis. To predict the immune response of NPC patients, patients were divided into response and no response group with TIDE value and chi-square test revealed that low m6A score group might have better response to immunotherapy (**Figure 7C**). Correlation analysis also showed that m6A score had obvious negative correlation with immune checkpoint molecules (**Figure 7D**). Previous study reported that the activation of NF-κB pathway played an important role in NPC (30, 31) and deletion of several NF-κB pathway components and cell cycle inhibitors such as CYLD, TRAF3, CDKN2A, and CDKN2B were found (18). After reanalyzing the copy number and mutation data provided in GSE102349, we confirmed that the deletion frequencies of NF-κB pathway components and cell cycle inhibitors were higher in high m6A score group, but the mutations were uncommon in general in genes in cell cycle, NF-κB or PI3K/MAPK pathways (**Figure 7E**). Considering activated cell cycle and that NF-κB pathways were reported to be highly associated with cancer stem cells (32, 33), mRNAsi, the mRNA based stemness index, was obtained to assess the stemness of NPC in GSE102349 (**Figure 7F**). The results showed mRNAsi was higher in high m6A score group, TME based subtype I, patients with progression, and differentiated histological type. Correlation analysis further confirmed that m6A score had a strong positive correlation with mRNAsi (**Figure 7G**) and this kind of correlation could also be found between m6A regulators and mRNAsi (**Figure 7H**). All the above evidence illustrated that low m6A score group with low progression probability might be associated with higher immune cell infiltration and better response to immunotherapy, and high m6A score group with high possibility of metastasis might possess more activated NF-κB pathway and higher cancer stemness index.

### m6A Modification Patterns in Pan-Cancer and the Predictive Role in Immunotherapy Efficacy

**Figure 8:**
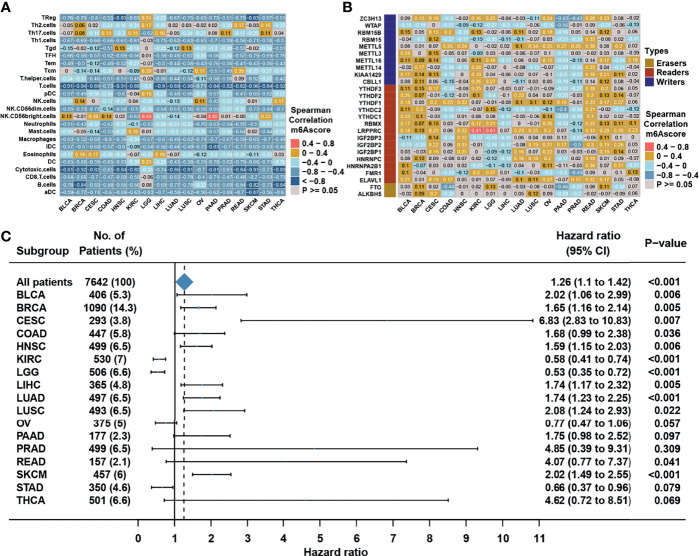
m6A modification patterns in pan-cancer. **(A)** Correlation analysis between m6A score and TME infiltration cell assessed by immune cell signature 1 using Spearman analysis in the TCGA pan cancer. **(B)** Correlation analysis between m6A score and m6A regulators using Spearman analysis in the TCGA pan cancer. **(C)** Subgroup analyses estimating prognostic value of m6A score in different cancer types from the TCGA datasets. HR >1.0 indicated that high m6A score was an unfavorable prognostic factor.

**Figure 9:**
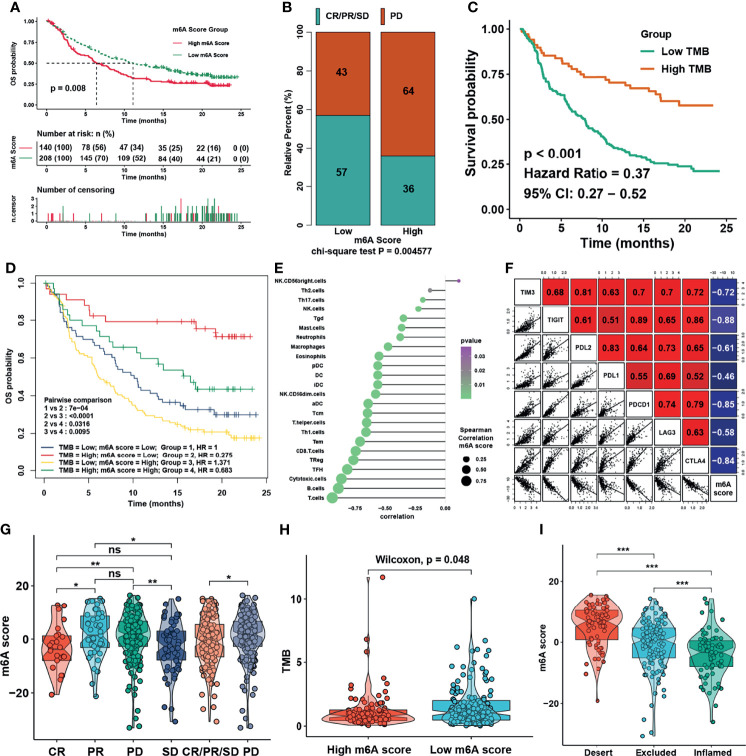
m6A modification patterns in the role in immunotherapy. **(A)** Survival analysis for m6A score in IMvigor210 cohort. **(B)** Rate of clinical response to anti-PD-L1 immunotherapy in high or low m6A score groups in the IMvigor210 cohort using chi-square test. **(C)** Survival analysis for TMB in the IMvigor210 cohort. **(D)** Survival analyses for patients treated with anti-PD-L1 immunotherapy stratified by both m6A score and tumor mutation burden (TMB) using Kaplan–Meier curves. **(E)** Correlations between m6Ascore and TME infiltration in NPC using Spearman analysis. **(F)** Correlations between m6Ascore and immune checkpoint inhibitors in NPC using Spearman analysis. **(G)** Distribution of m6A score in distinct anti-PD-L1 clinical response groups. **(H)** Distribution of TMB in different m6A score groups. **(I)** Differences in m6A score among distinct tumor immune phenotypes in IMvigor210 cohort. The asterisks represented the statistical p-values in panels **(F, H)** (*P < 0.05; **P < 0.01; ***P < 0.001); ns, no significance.

We then set to verify whether the m6A score could reflect the immune cell infiltration levels and its prognostic values in predicting response to immunotherapy. However, due to the lack of cohorts treated with immunotherapy with public matrix profile in NPC, we could only firstly illustrate the general applicability of m6A score in pan-cancer instead. Correlation analysis between m6A score and immune cell infiltration levels was conducted using ssGSEA with immune cell signature 1 in pan-cancer (17 types of cancer included) in the TCGA database. The result showed that m6A score was negatively correlated with most of immune cells such as DCs, T cells, and B cells in pan-cancer (**Figure 8A**). Correlation analysis between m6A score and m6A regulators in pan-cancer also demonstrated that m6A score could reflect the expression level of m6A regulators in pan-cancer (**Figure 8B**), and the candidate m6A regulator LRPPRC had obvious positive correlation with m6A score in almost all cancer types. Although the results in pan-cancer analysis were heterogeneous, m6A score was identified as an unfavorable prognostic biomarker in 17 types of independent cancers in the TCGA cohorts (**Figure 8C**). Based on that, we further validated the predictive function of m6A score in response to immune checkpoint inhibitors in an anti-PD-L1 cohort (IMvigor210) in urinary carcinoma, which was an alternative cohort with consistent conclusions from the TCGA bladder cancer (BLCA) cohort. Survival analysis showed patients with low m6A score presented better prognosis and clinical response to anti-PD-L1 than patients from high m6A score group (**Figures 9A, B**). Patients with higher tumor mutation burden (TMB), a biomarker closely linked to immunotherapeutic efficacy, had better prognosis than patients with lower TMB (**Figure 9C**), and patients with a combination of low m6A score and high TMB presented the greatest survival advantage (**Figure 9D**). Correlation analysis showed m6A score was also negatively correlated with immune cell infiltration (**Figure 9E**) and expression of immune checkpoint molecules (**Figure 9F**). It could be found that m6A score in complete response (CR) group was lower than those in partial response (PR) and progressive disease (PD) groups (**Figure 9G**). Wilcoxon test showed that TMB in low m6A score group was higher than that in high m6A score group (**Figure 9H**), which provided additional evidence for the function of m6A score in predicting immunotherapy response. Moreover, higher m6A score was associated with desert and excluded immune phenotypes, in which it was difficult for ICIs to exhibit the desired antitumor activities (**Figure 9I**). The candidate m6A regulator LRPPRC was significantly negatively correlated with m6A score (**Figure S4A**) and was a risk factor in the anti-PD-L1 dataset (**Figures S4B–C**). Furthermore, LRPPRC expression was obviously positively correlated with Th2 cell infiltration while negatively correlated with most of other immune cell infiltrations (**Figure S4D**). Taken together, this study indicated that m6A modification patterns were significantly correlated with tumor immune infiltrations and response to immunotherapy, and the established m6A score had a potential to predict the response to immunotherapy.

## Discussion

The evolving landscape of m6A modification in the tumor microenvironment provoked our interest in the role of m6A in NPC. However, different from most studies focusing on single m6A regulator or single immune cell type, the present study comprehensively recognized the overall immune cell infiltration characterizations mediated by integrated roles of multiple m6A regulators. Based on 26 m6A regulators, two distinct m6A modification subtypes with distinct TME cell infiltration characterizations were constructed. Based on differentially expressed genes between m6A subtypes, m6A gene clusters and m6A score were further identified, revealing that the mechanism underlying the carcinogenesis and metastasis of NPC was associated with immune cell infiltration, EBV infection, and cancer stemness index. Subtype 1, cluster 1, and low m6A score group were characterized by the activation of innate and adaptive immunity, corresponding to immune activated phenotype, which had numerous immune cell infiltration in TME (34–36); while subtype 2, cluster 2, and high m6A score group were characterized by immune suppression, corresponding to immune suppressed phenotype, which lacked activated and primed T cell (37). Most importantly, m6A score was significantly negatively correlated with immune checkpoint molecules, CD8 T cell effector, and antigen processing machinery, and was validated to have prognostic value and negative correlation with TME infiltration in pan-cancer. The results of the study suggested that m6A score was a reliable tool for comprehensively evaluating the m6A modification patterns of individual tumor, and further determining the TME infiltration patterns and also predicting the response to immunotherapy.

Some studies had reported the role of m6A modification in NPC, and for instance, METTL3 could aggravate the progression of NPC through mediating Snail or EZH2 (38, 39). However, the present study for the first time depicted m6A regulators landscape *via* integrated analysis, and found that they were heterogeneously expressed in NPC and that the most possible mechanism for the differential expression of these genes was DNA methylation degree. Almost all m6A regulators had different levels of DNA methylation in the promoter region, while somatic mutation and copy number variation of m6A regulators were not obvious and prevalent. The correlation analysis showed that expressions of erasers FTO and ALKBH5 were highly negatively associated with those of the writers and readers, while the expressions of writers and readers were positively correlated with each other. It is worth mentioning that the candidate m6A regulator LRPPRC, leucine-rich PPR-motif-containing protein, was consistently highly expressed in NPC when compared with normal nasopharyngeal epithelial tissue or cells in several NPC datasets and it was also a risk factor for PFS. In addition, LRPPRC could reflect the level of m6A score and mRNAsi. More importantly, LRPPRC was significantly positively correlated with EBV gene and immune suppressive Th2 cells, but negatively correlated with immune checkpoint molecules and most of immune activated cell types. Previous studies showed that LRPPRC could negatively regulate mitochondrial antiviral signaling during hepatitis C virus infection (40) and could suppress genome instability and hepatocellular carcinomas by sustaining Yap-P27-mediated cell ploidy and P62-HDAC6-mediated autophagy maturation (41). Taken together, it is reasonable to speculate that LRPPRC-mediated m6A methylation may suppress the activation of TME infiltration and facilitate EBV infection, thus inhibiting intra-tumoral antitumor immune response.

NPC is a malignant tumor with good prognosis, and local recurrence and distant metastasis has been the main cause of death in the IMRT era (42). The constructed m6A score in this study could well predict the risk of metastasis and reflect the previously defined clinical stage and TME subtype (18). It is true that the TME subtype could also reflect the TME infiltration and PFS in NPC, but the construction method of m6A score was completely different and the functions between m6A score and TME subtype were partially distinctive. In terms of the method, the PCA algorithm adopted in this study could retain the most characterization of m6A regulators and that was why m6A score displayed a high association with most m6A regulators. Here, we used m6A gene clusters to further fix the inaccuracy of m6A subtypes, and then used m6A score for dimensionality reduction of m6A gene clusters and m6A subtypes. Unlike m6A subtypes or m6A clusters which are categorical variables, the m6A score was a specific numerical variable through PCA algorithm conversion, which could be used to conduct correlation analysis with other numerical variables, such as infiltration levels of immune cells, mRNAsi, and the expression of molecules. In terms of the function, the m6A score closely correlated with the function of cancer stem-like cells (CSCs) and NF-κB signaling pathway. CSCs are hypothesized to be the key factor in cancer metastasis and recurrence (43). Calculation of mRNAsi also revealed that m6A score was positively correlated with mRNAsi, which could be the reason why m6A score was a risk factor for metastasis. Moreover, copy number deletion of many NF-κB signaling pathway inhibitors and copy number amplification of many NF-κB signaling pathway activators could also be related with CSCs and poor prognosis. Multiple studies have indicated that immunotherapy could shed light on developing safer and more effective treatment modalities for NPC in the future (44–46). According to our analysis, both m6A score and most m6A regulators were negatively correlated with immune checkpoint molecules such as TIM3, TIGIT, PDL1, PD1, CTLA4, and LAG3. When combined with the results of TME infiltration, the function of m6A score and m6A regulators in immunotherapeutic efficacy could be reasonable and obvious. Previous study has revealed that EBV infection and the expression of latent EBV genes are postulated to drive the tumorigenesis of NPC through multiple pathways (47). Although we could not get the original expression of EBV gene, based on GSVA analysis we robustly assessed the EBV gene expression in NPC in GSE102349, and found that EBV gene expression was as expected negatively correlated with “cold” TME infiltration while positively correlated with mRNAsi. The result is closely consistent with a recent study reporting that m6A protein YTHDF1 could suppress EBV replication and promote EBV RNA decay (48), which also indicated that m6A could play a pivotal role in anti-EBV process in NPC.

### Conclusions

In conclusion, the m6A score had a great potential to comprehensively evaluate the m6A methylation modification patterns and their corresponding immune cell infiltration characteristics in TME. It could also be used to evaluate the clinicopathological features including clinical stages, tumor differentiation levels, TME subtypes, genetic variation, EBV infection, and mRNAsi of individual patient, and also further inferring the immune phenotypes in tumors and predict the survival and clinical response to immunotherapy of patients. More importantly, this study provided insights in the development of novel immunotherapeutic agents specifically targeting m6A regulators or m6A phenotype-related genes to further reverse the TME cell infiltration characterization into “hot tumors”, thus improving the response to immune checkpoint inhibitor.

## Data Availability Statement

The datasets presented in this study can be found in online repositories. The names of the repository/repositories and accession number(s) can be found in the article/**Supplementary Material**.

## Author Contributions

ZL and JHe designed this work. ZL, JHe, WL and JHa integrated and analyzed the data. JY and WL collected and preprocessed the data. ZL and JHe wrote this manuscript. JHe and NC edited and revised the manuscript. All authors contributed to the article and approved the submitted version.

## Conflict of Interest

The authors declare that the research was conducted in the absence of any commercial or financial relationships that could be construed as a potential conflict of interest.

## Publisher’s Note

All claims expressed in this article are solely those of the authors and do not necessarily represent those of their affiliated organizations, or those of the publisher, the editors and the reviewers. Any product that may be evaluated in this article, or claim that may be made by its manufacturer, is not guaranteed or endorsed by the publisher.
