# Supplementary material for: m6A Regulators Mediated Methylation Modification Patterns and Tumor Microenvironment Infiltration Characterization In Nasopharyngeal Carcinoma
Source: Front Immunol. 2022 Jan 7;12:762243. doi: 10.3389/fimmu.2021.762243 (PMC8776994; doi:10.3389/fimmu.2021.762243)
Supplement: Supplementary file 1 [file DataSheet_1.pdf]

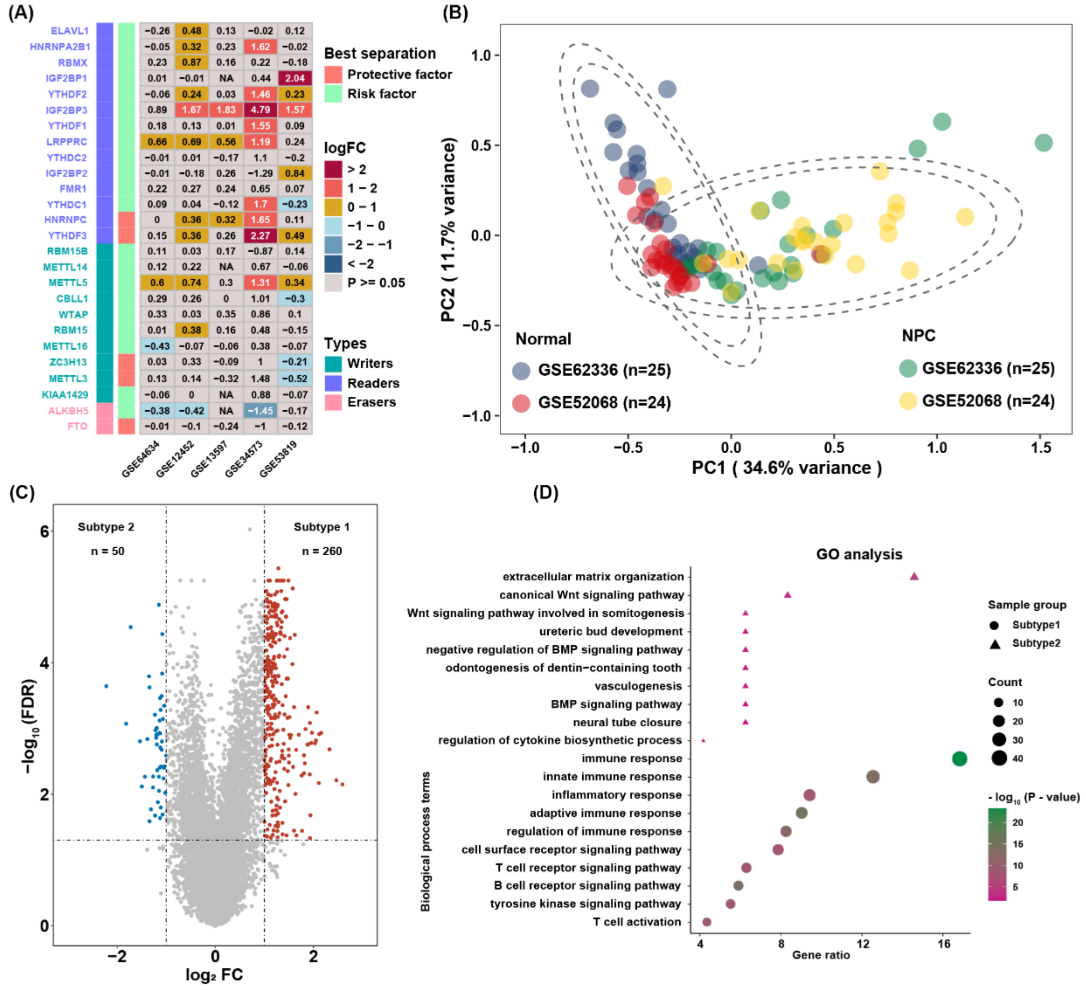

Figure S1. Differentially expressed analysis of mRNA and DNA methylation for m6A regulators and prognostic analysis. (A) Differentially expressed analysis of m6A regulators in five independent datasets. (B) Principal component analysis for the DNA methylation of m6A regulators in two independent datasets, showing a remarkable difference on DNA methylation level between normal tissues and NPC. (C) Volcano plot showed the differentially expressed genes between m6A subtype 1 and 2, including 260 genes highly expressed in subtype 1 and 50 genes highly expressed in subtype 2. (D) GO analysis of differentially expressed genes between m6A subtypes and GO term biological function was shown.

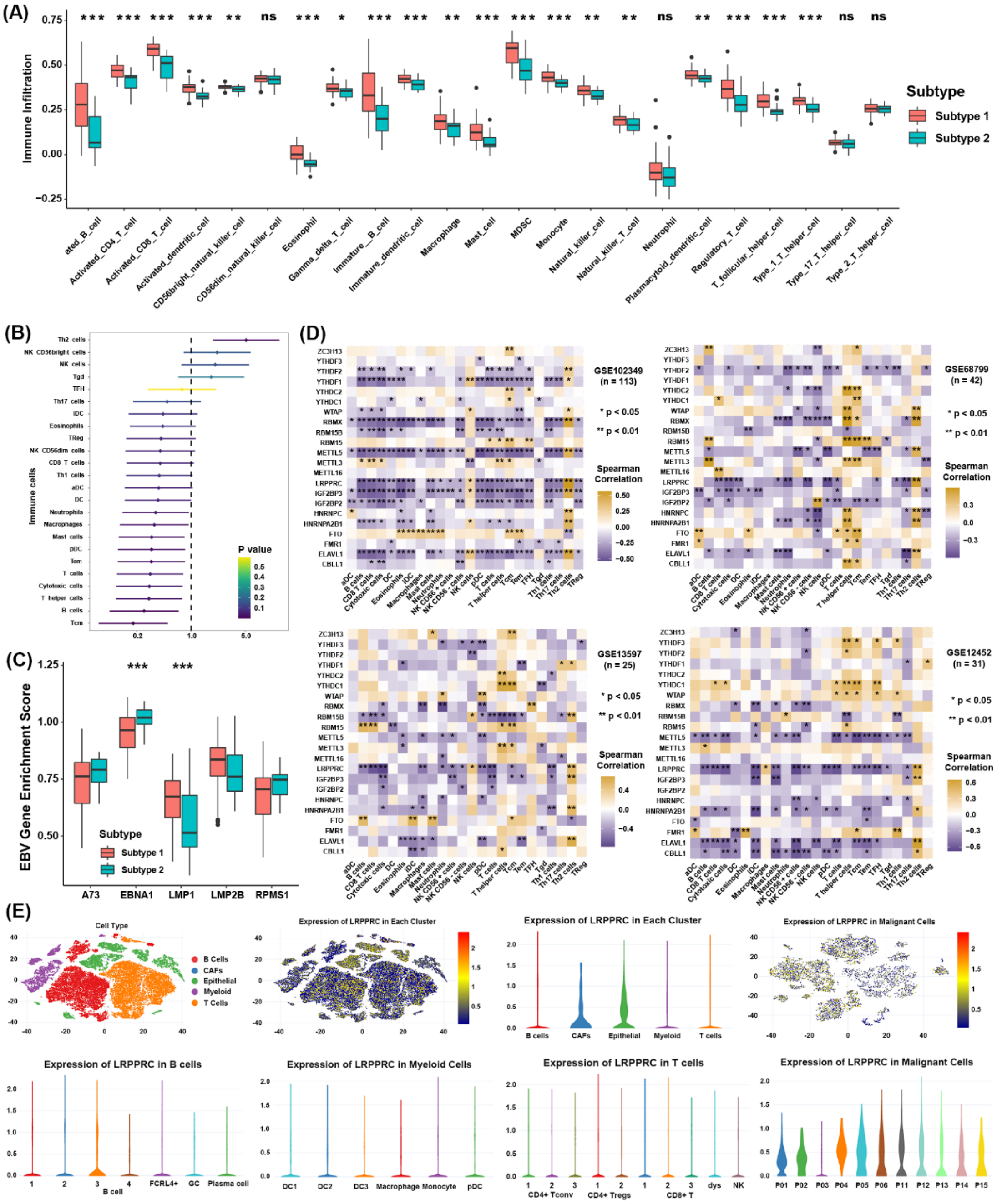

Figure S2. TME infiltration characteristics of m6A subtypes. A The abundance of GSVA score for TME infiltration cell by immune cell signature 2 in m6A subtypes. B The prognostic analyses for TME infiltration cell assessed by immune cell signature 1 in GSE102349 cohorts, and hazard ratio >1 represented risk factor for survival and hazard ratio <1 represented protective factor for survival. C The abundance of EBV gene enrichment score by GSVA in m6A subtypes. D Correlation analysis between

TME infiltration cell assessed by immune cell signature 1 and 26 m6A regulators in four dependent NPC datasets. E Expression of candidate m6A regulator LRPPRC in single layer including B cells, T cells, CAFs, Epithelial and myeloid cells. The asterisks represented the statistical p value (*P < 0.05; **P < 0.01; ***P < 0.001).

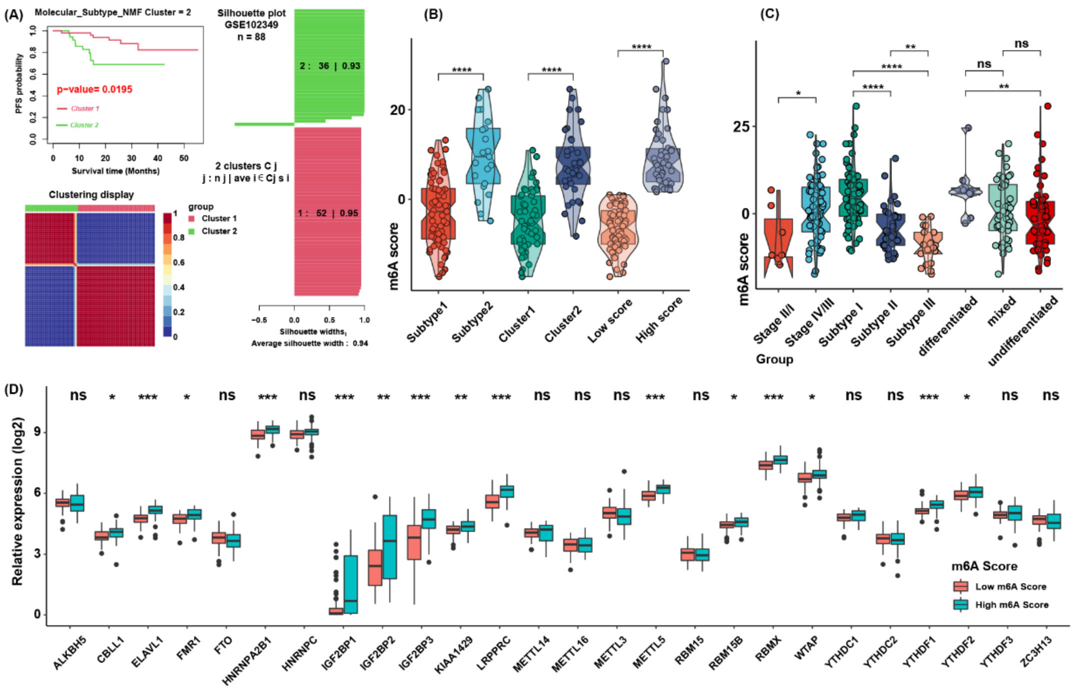

Figure S3. NMF algorithm to construct m6A clusters and clinical characteristics of m6A score. A The result of NMF algorithm for k = 2 with the best average silhouette width 0.94. B The abundance of m6A score in m6A subtype, m6A clusters and m6A score groups. C The abundance of m6A score in clinical stages, TME subtypes and morphology. D The abundance of m6A regulators in different m6A score groups. The asterisks represented the statistical p value (*P < 0.05; **P < 0.01; ***P < 0.001).

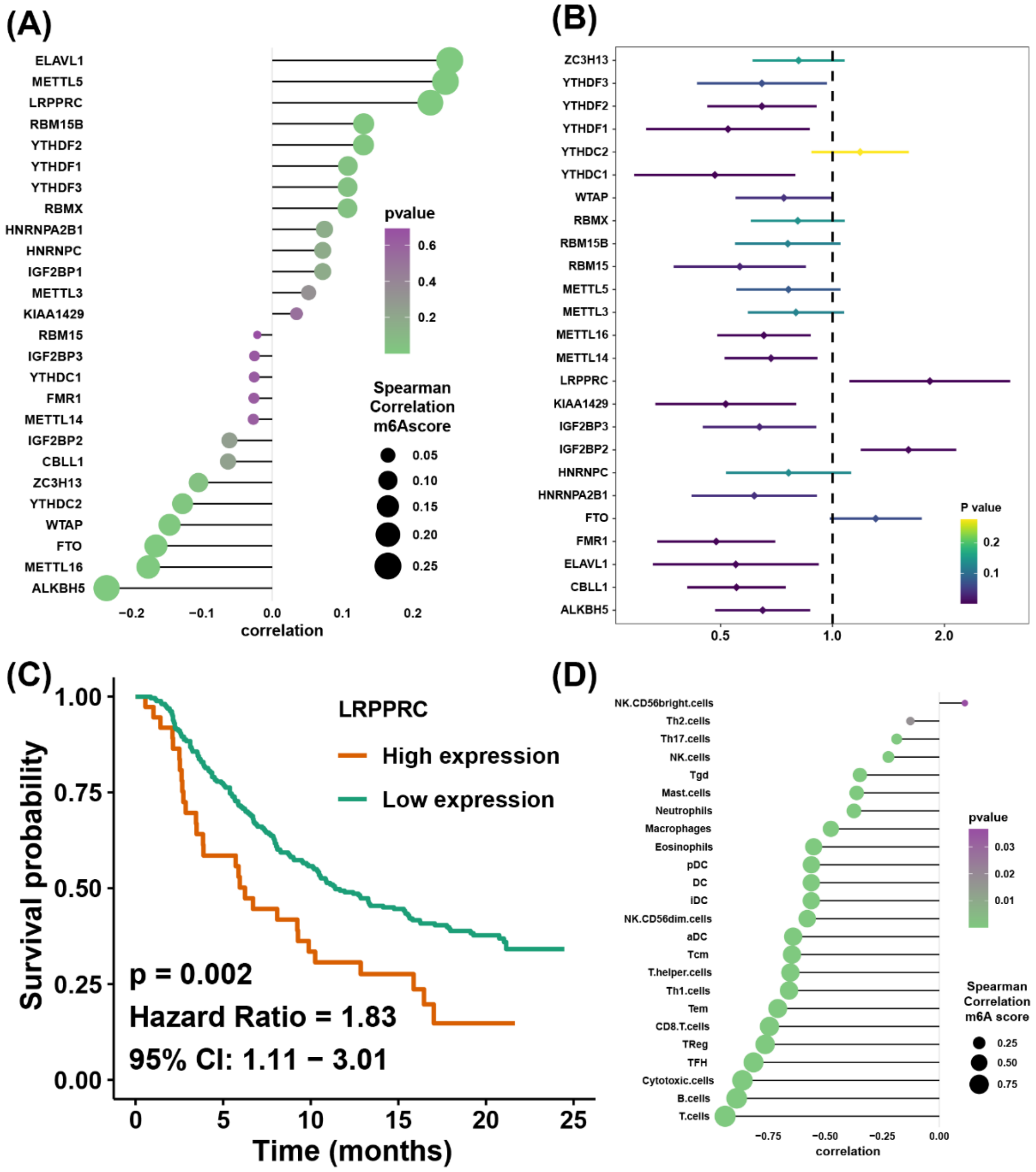

Figure S4. The role of LRPPRC in immunotherapy. (A) Correlation analysis between m6A score and m6A regulators using Spearman analysis in the IMvigor210 cohort. (B) Hazard ratios of m6A regulators in anti-PD-L1 cohort IMvigor210. (C) Survival analysis for candidate m6A regulator LRPPRC in the IMvigor210 cohort. (D) Correlation analysis between expression of LRPPRC and TME infiltration cell using Spearman analysis in the IMvigor210 cohort.
